# Enhancing Karate Performance: Development and Validation of a Karate-Specific Change-of-Direction Test

**DOI:** 10.3390/jfmk10040417

**Published:** 2025-10-23

**Authors:** Said Ben Hassen, Raouf Hammami, Yassine Negra, Senda Sammoud, Roland van den Tillaar

**Affiliations:** 1 Higher Institute of Sport and Physical Education of Ksar-Said, University of Manouba, University Campus, Manouba 2010, Tunisia; karasaidn@yahoo.fr (S.B.H.); raouf.cnmss@gmail.com (R.H.); yassinenegra@hotmail.fr (Y.N.); senda.sammoud@gmail.com (S.S.); 2 Research Laboratory (LR23JS01) “Sport Performance, Health & Society”, Higher Institute of Sport and Physical Education of Ksar-Said, University of Manouba, University Campus, Manouba 2010, Tunisia; 3 Tunisian Research Laboratory “Sports Performance Optimization”, National Center of Medicine and Science in Sports (CNMSS), Tunis 1003, Tunisia; 4 Department of Sports Sciences and Physical Education, Nord University, 7600 Levanger, Norway

**Keywords:** karate athletes, performance assessment, athletic conditioning, sport-specific testing, physical fitness evaluation, athlete profiling, training optimization, change-of-direction speed measurement

## Abstract

**Background:** Change-of-direction (CoD) ability is critical in karate, yet sport-specific assessment tools are limited. This study aimed to evaluate the reliability and validity of a newly developed karate-specific CoD test for male and female athletes and to examine its relationships with other motor performance measures. **Methods:** Thirty-six experienced karatekas (20 men: age 20.8 ± 1.8 years, height 1.79 ± 0.05 m, body mass 73.1 ± 10.6 kg; 16 women: age 21.2 ± 1.7 years, height 1.67 ± 0.04 m, body mass 63.5 ± 8.9 kg), all national squad members, participated. Athletes performed the karate CoD test twice to assess test–retest reliability, and completed additional CoD tests (Y CoD, T-half), linear sprint tests, standing long jump, and Y-balance tests to evaluate criterion validity and associations with other motor abilities. **Results:** The karate CoD test demonstrated excellent reliability (ICC = 0.996), with similar consistency in men and women. Criterion validity was supported by a meaningful correlation with the Y CoD test, but not with the T-half test. Associations with linear sprinting and lower-limb power (standing long jump) were weak or inconsistent, indicating that karate-specific CoD performance is distinct from general physical capacities. **Conclusions:** The karate CoD test shows good reliability and validity for assessing planned change-of-direction ability in elite karate athletes. Its use should be limited to pre-planned movements and complemented with other tests (e.g., reactive agility, sprinting, jumping, strength) for a fuller performance assessment.

## 1. Introduction

Performance in karate relies on the integration of high physical fitness, technical proficiency, and tactical intelligence [1]. The sport is characterized by rapid offensive and defensive maneuvers, requiring frequent forward and backward movements, lateral displacements, hopping, and rotational actions performed over short distances within a confined combat space [2]. These dynamic and multidirectional actions place a premium on change-of-direction (CoD) ability, enabling athletes to decelerate, accelerate, and reorient efficiently in response to anticipated movement patterns and tactical demands [3,4].

It is important to distinguish between CoD and agility, as the two constructs are often conflated. Agility encompasses both the physical capacity to change direction and the perceptual–cognitive processes needed to respond to unpredictable stimuli, such as an opponent’s attacks or feints [1,5]. In contrast, CoD refers specifically to pre-planned movements involving rapid deceleration, acceleration, and body reorientation. The present study focuses on CoD, as the newly developed test evaluates pre-determined, sport-specific movement sequences rather than reactive responses, ensuring conceptual precision and alignment with its intended purpose.

Although CoD has been widely studied in team sports [5,6,7,8,9], limited research has examined its unique biomechanical and perceptual-motor demands in karate. The sport requires frequent stance transitions, rapid weight shifts, and explosive repositioning in response to opponent movements and timing [1,2]. Generic CoD assessments, such as the Illinois or T-test [8], lack ecological validity for karate because they overlook its spatial-temporal, technical, and perceptual-motor characteristics [10,11]. Previous sport-specific adaptations, such as the Modified Illinois CoD Test for soccer [12], demonstrate the importance of designing assessments that reflect sport-specific movement and tactical demands. However, earlier karate-focused efforts [13] did not fully integrate the distinctive movement sequences, stance dynamics, and technical actions inherent to competitive karate. Developing and validating a karate-specific CoD test therefore fills a critical gap, providing a more ecologically valid and practically relevant assessment tool.

From a theoretical perspective, such a test should be guided by the ecological dynamics approach, emphasizing perception–action coupling in skill execution [14], and the principle of motor specificity, which proposes that testing and training tasks should replicate the spatial-temporal and technical constraints of the sport [10]. A validated karate-specific CoD test would allow practitioners to monitor sport-specific performance, identify inter-limb asymmetries, track progression, and design individualized training interventions, while also supporting talent identification and long-term athlete development [8,9].

Accordingly, the present study had two primary objectives: (1) to establish the reliability and validity of a newly developed karate-specific CoD test, and (2) to examine its relationships with other key components of athletic performance. By addressing these objectives, this study aims to provide a scientifically grounded, sport-specific assessment tool that can support evidence-based monitoring, training optimization, and performance enhancement in karate.

## 2. Materials and Methods

This observational cross-sectional study was designed to assess the reliability (test–retest) and criterion validity of a newly developed karate-specific change-of-direction (CoD) test. The karate CoD test was developed to replicate the fundamental movements and skills typically performed during karate competition. Criterion validity was examined by comparing performance in the karate CoD test with established generic CoD assessments (T-half and Y CoD tests). Test–retest reliability was evaluated through two trials conducted one week apart. In addition, sprinting, jumping, and dynamic balance performances were measured to examine their associations with karate CoD performance.

### 2.1. Participants

A total of thirty-six karatekas (20 men: age = 20.8 ± 1.8 years, height = 1.79 ± 0.05 m, body mass = 73.1 ± 10.6 kg; 16 women: age = 21.2 ± 1.7 years, height = 1.67 ± 0.04 m, body mass = 63.5 ± 8.9 kg), all members of the Tunisian national karate team, volunteered to participate in this study. Participants were considered experienced karate practitioners, having maintained a high-level training regimen over the previous five years, consisting of eight to nine sessions per week lasting approximately 80–90 min each.

A convenience sampling method was used, as all athletes were recruited from the national squad based on availability during the testing period. Inclusion criteria required participants to be active national team members, free from musculoskeletal injuries, medical conditions, or pain that could affect performance, and to have a minimum of five years of continuous karate training. Exclusion criteria included recent injuries (within the past six months), chronic medical conditions, or inability to complete all testing procedures. This approach ensured a homogeneous, elite-level sample suitable for test validation while controlling for potential confounding factors, though it may limit generalizability to non-elite or youth karate populations.

Based on international ranking, 25 karatekas (20 men and 5 women) were classified as *high-ranked*, having competed in World Championships and holding international WKF standings, while the remaining 11 (8 women and 3 men) were classified as *low-ranked*, having primarily participated in national or regional competitions. Prior to participation, all athletes (and their legal guardians, when applicable) provided written informed consent. The study was conducted in accordance with the latest version of the Declaration of Helsinki, and the protocol was approved by the Local Ethics Committee of the National Centre of Medicine and Science of Sports of Tunis (CNMSS-LR09SEP01, on 20 November 2024).

### 2.2. Procedure

This study was conducted during the competitive season, and participants completed three familiarization sessions prior to testing to ensure proficiency with all protocols. The first session focused on anthropometric measurements, including height and body mass, while the subsequent sessions allowed participants to practice all performance tests under standardized conditions. All testing was performed within a two-week period, with a minimum of 48 h between sessions to minimize fatigue effects. Testing sessions were conducted at the same time of day (±1 h) for each participant to control for diurnal variations, in an indoor sports hall with consistent temperature and lighting conditions. Participants were instructed to maintain normal hydration and dietary habits and to avoid intense training or strenuous activity 24 h prior to each testing session.

### 2.3. Measurements

**Figure 1:**
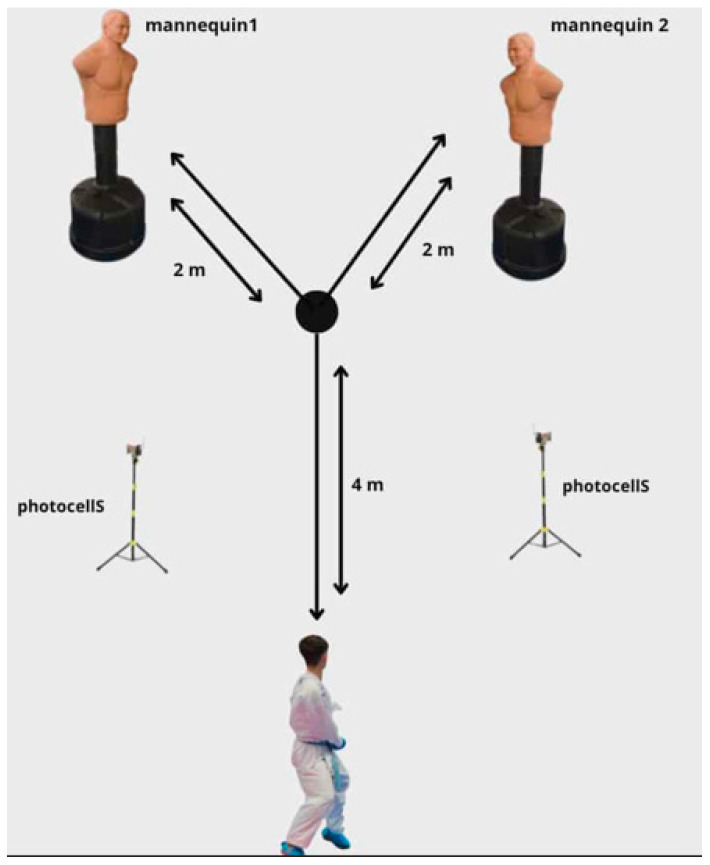
Schematic of the karate-specific test.

The dimensions, layout, and movement pathway of the karate change-of-direction (CoD) test are illustrated in Figure 1. Participants began the test from the start line and performed the following sequence: (1) move quickly to the center point while maintaining a guard position without crossing the feet; (2) perform a lateral shift toward mannequin 1 and execute an Oi Tsuki punch; (3) return to the center point; (4) advance in a guard position toward mannequin 2 and execute a Mawashi Geri kick; (5) retreat backward to the center point; and (6) return to the start/finish line while maintaining the guard position.

Performance time was recorded using electronic photocell timing gates (Brower Timing Systems, Salt Lake City, UT, USA) positioned 0.75 m above the ground, with an accuracy of 0.001 s. Timing started when participants crossed the start line and stopped upon their return. Trials were repeated following a three-minute rest interval if any step or technique was judged technically incorrect by certified evaluators. To minimize subjectivity, two independent karate coaches assessed technical execution, and inter-rater agreement was established prior to data collection to ensure consistent judgments. The best time from three valid trials on each test day was retained for analysis, with a minimum of two minutes of passive recovery between trials.

The test was developed to replicate the essential biomechanical and tactical elements commonly observed in competitive karate, including rapid forward–backward transitions, lateral displacements, stance adjustments, and the integration of offensive techniques under spatial constraints [2]. Although the present study did not perform a formal kinematic or video-based validation, the design of the test was directly informed by detailed analyses of competition footage and expert consultation with national-level karate coaches, who confirmed that the movement patterns and execution sequence closely mirror those used during match play. This expert-informed development process provides preliminary support for the test’s ecological relevance to karate performance. Nonetheless, we acknowledge that direct empirical validation remains necessary. Future studies should include motion-capture or video-based analyses comparing the kinematics and temporal structure of test movements to those observed in actual competitive contexts to further substantiate the test’s ecological validity.

Two established change-of-direction tests were used as reference measures to assess the criterion validity of the newly developed karate CoD test: the T-half and the Y CoD tests. The T-half test was conducted following the protocol of Sassi et al. [11]. This shorter variation in the traditional T-test requires forward sprinting, lateral shuffling, and backpedaling. Timing gates (Brower Timing Systems, Salt Lake City, UT, USA) were positioned 0.75 m above the ground at the start/finish line. Timing started when participants broke the start beam and stopped when they crossed the finish beam after backpedaling. The T-half test has been shown to be a valid and reliable tool for assessing CoD performance, particularly in contexts where the full T-test may be too demanding or less sport-specific.

The Y CoD test was administered according to Dellal et al. [12]. Participants sprinted 5 m through a timing gate to trigger trial initiation, performed a 45° cut to the left or right, and then sprinted an additional 5 m through the target gate where timing stopped, for a total distance of 10 m. An electronic timing system (Brower Timing Systems, Salt Lake City, UT, USA) with 0.001 s accuracy was used, with gates positioned 0.75 m above the ground to detect trunk passage. The fastest time of three attempts was recorded for analysis.

In addition to these criterion measures, other performance tests were conducted to examine associations between karate CoD performance and general motor abilities, including sprinting, jumping, and dynamic balance. Linear sprint performance was measured over 10 m with split times recorded at 5 m using a single-beam electronic timing system. Three timing gates were positioned 0, 5, and 10 m from the start line, 0.75 m above the ground to detect trunk passage. Participants started from a standing split stance, with the lead foot placed 0.3 m behind the start gate.

Jumping performance was assessed using the bilateral Standing Long Jump test. Participants began with feet shoulder-width apart behind a marked line, flexed their legs and arms to propel forward, and landed with both feet simultaneously while maintaining balance. The horizontal distance from the starting line to the heel of the rear foot was recorded to the nearest centimeter [15].

Dynamic balance was evaluated using the lower-quarter Y-balance test, following [16]. Participants performed the test barefoot to minimize shoe-surface confounding effects, standing on their dominant leg and reaching with the opposite leg in three directions: anterior, posteromedial, and posterolateral. Trials were repeated if specific criteria were not met, including loss of balance or improper foot placement. Three successful trials were recorded for each leg, and the best performance was retained. Leg length was measured from the anterior superior iliac spine to the distal medial malleolus [17], and the composite score (CS) was calculated as: CS = [(maximum anterior reach distance + maximum postero-medial reach distance + maximum postero-lateral reach distance)/(leg length × 3)] × 100 [18].

Furthermore, all test trials were monitored by two certified karate coaches who independently evaluated the technical correctness of each movement and strike execution. Disagreements were discussed and resolved immediately to maintain consistency in trial validation. Before data collection, both evaluators participated in a calibration session using pilot video recordings to standardize assessment criteria. Inter-rater reliability for these technical judgments was later quantified using Cohen’s kappa coefficient (κ), based on a random subset of 30 recorded trials.

### 2.4. Statistical Analysis

JASP v.9.5.1 (University of Amsterdam, Amsterdam, the Netherlands) software was employed for data analysis, with a significance criterion set at *p* < 0.05 for all tests. Descriptive statistics, encompassing mean and standard deviation, were utilized for data presentation. The normality of distribution across all variables was assessed using the Shapiro–Wilk test [19].

To explore potential learning effects or systematic biases between karate CoD test and retest scores, a dependent samples *t*-test was conducted. Relative and absolute reliability were evaluated using the Intraclass Correlation Coefficient (ICC_1,3_3) and the typical error of measurement (TEM), respectively. ICC values below 0.40 were deemed poor, between 0.40 and 0.70 moderate, between 0.70 and 0.90 good, and ≥0.90 excellent [20].

In addition to the intraclass correlation coefficient (ICC) and coefficient of variation (CV), Bland–Altman analyses were conducted to assess the level of agreement between test and retest measurements. The mean bias (systematic error) and 95% limits of agreement (LOA) were calculated according to Bland and Altman [21]. Bland–Altman plots were constructed to visually inspect the distribution of differences across the measurement range and to identify any potential heteroscedasticity or outliers.

The Minimal Detectable Change at the 95% confidence interval (MDC_95_) was computed using the formula: MDC_95_ = TEM × 1.96 × √2 [22]. An independent *t*-test was performed for all tests to identify of there was a sex effect. Cohen’s d was classified as trivial (0.00 ≤ d ≤ 0.19), small (0.20 ≤ d ≤ 0.49), medium (0.50 ≤ d ≤ 0.79), and large (d ≥ 0.80) [23]. To assess associations between the karate CoD test with the other motor abilities for men and women linear regressions (Pearson’s correlations) were compared. To assess sex effect, the regression lines on the two sex groups were compared with the regression on the pooled data of males and females using an F-test as described by Crowder and Hand [24]. Correlation coefficients ranging from 0 to 0.1 are considered “trivial”, 0.11 to 0.33 “small”, 0.31 to 0.5 “moderate”, 0.51 to 0.7 “large”, 0.71 to 0.9 “very large”, and 0.9 to 0.99 “nearly perfect”. Coefficients of determination (R^2^) were utilized to quantify the explained variance between tests according to Weir [25].

## 3. Results

### 3.1. Validity and Reliability of Karate CoD Test

All parameters were normally distributed as shown be the Shapiro–Wilk test (*p* > 0.05). The mean times of the karate CoD test were 7.65 ± 0.48 (test) and 7.64 ± 47 s (retest) with no significant differences between the two tests (*p* = 0.12). The ICC [95% CI] was 0.996 between the test and retest scores with a TEM of 0.16 s (2.13%, Table 1).

**Figure 2:**
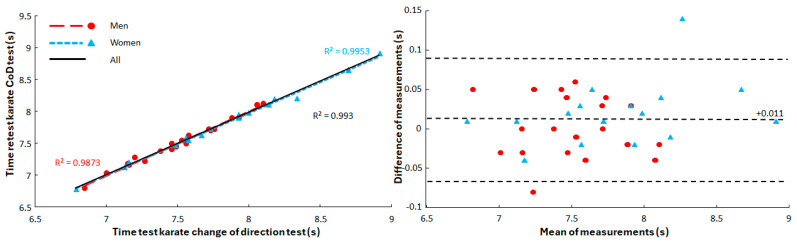
Test and retest times of the karate change-of-direction test with correlation and R^2^ for men, women and all together and Bland–Altman plot on mean and differences between test and retest performances. Bland-Altman plot with 95% confidence intervals (dashed lines).

The men performed significantly better than women in the specific change-of-direction speed test (t = −2.063, *p* = 0.047, d = 0.69). The R^2^ for men, women and all were ≥0.987 with no significant sex differences between the correlation curves for men and women with the pooled one (*p* = 0.276, Figure 2).

Bland–Altman analyses demonstrated minimal systematic bias between test and retest measurements (0.011 s), with mean differences close to zero and narrow 95% limits of agreement (Figure 2). The visual inspection of the plots confirmed a random distribution of residuals across the measurement range, indicating an absence of proportional bias. These findings support the excellent reliability indicated by the ICC values. In addition, inter-rater reliability analysis demonstrated excellent agreement between the two evaluators (Cohen’s κ = 0.91, 95% CI [0.85–0.97]), confirming a very high level of consistency in identifying technically correct versus incorrect trials.

### 3.2. Correlations with Other Motor Abilities

**Figure 3:**
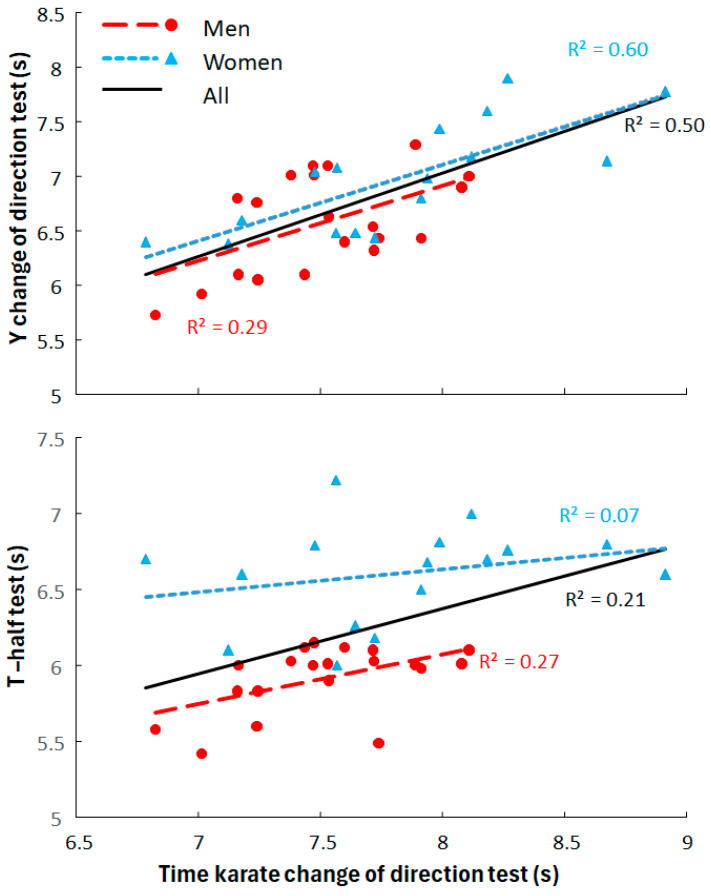
Correlations and R^2^ between time-specific change-of-direction speed test with T-half and Y change-of-direction tests for men and women and pooled one.

**Figure 4:**
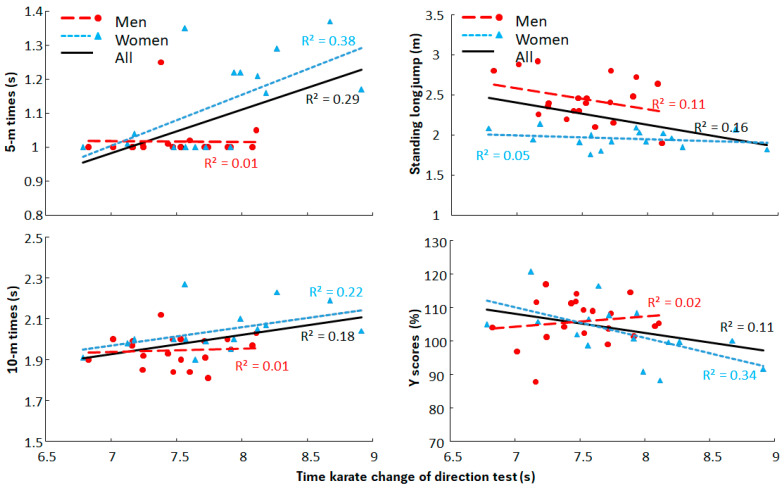
Correlations and R^2^ between time-specific change-of-direction speed test with 5 and 10 m sprint times, standing long jump and Y scores for men and women and pooled one.

Also significant differences were found between the results of men and women in the other motor ability tests (t ≤ −2.5, *p* ≤ 0.016, d ≥ 0.8, Figure 3 and Figure 4), except not for the Y scores (t = 1.25, *p* = 0.22, d = 0.419, Figure 4). Since the relationship between the test and retest were so high with low standard error of the mean the average times of the test and retests per subject were used and compared with the other motor abilities. The correlations between the karate CoD test with the other motor ability test showed that for men only significant moderate correlations with the other two CoD tests (r = 0.54 and r = 0.52) were observed, while in women significant high correlation were found with the Y CoD test (r = 0.77), and moderate correlations with 5 m sprint times (r = 0.61) and Y scores (r = −0.58). When the data for the men and women were pooled only a significant high correlation was observed with the Y CoD test (r = 0.71), while with the other motor abilities moderate correlations were found with the other motor abilities (0.34 ≤ r≤0.53) were observed (Figure 3 and Figure 4). However, when comparing the regressions on the pooled data with the men and women, significant different regression lines were found between these for T-half test (t = −3.0, *p* = 0.005) and y scores (t = 3.1, *p* = 0.004) with all low coefficient of determinations (0.11 ≤ R^2^ ≤ 0.29, Figure 3 and Figure 4).

## 4. Discussion

The purpose of this study was to evaluate the reliability and criterion validity of a karate-specific change-of-direction (CoD) test in male and female karate athletes and to examine its associations with other motor performance abilities. The findings indicate that the karate CoD test demonstrated a meaningful correlation with the Y CoD test, but not with the T-half test, while associations with linear sprinting and lower-limb power (standing long jump) were weak or inconsistent.

The stronger correlation with the Y CoD test can be explained by the similarity of movement characteristics. Both the karate CoD and Y CoD tests involve frequent multidirectional accelerations and decelerations combined with rapid pre-planned reorientations, reflecting sport-specific displacement patterns [6]. By contrast, the T-half test emphasizes fewer directional changes and longer straight sections, making it less sensitive to the fine-grained control, stance stability, and rapid body reorientation required in karate [11]. These findings support the construct validity of the karate CoD test as a measure of pre-planned, sport-specific CoD ability, rather than reactive agility, which additionally involves perceptual–cognitive decision-making.

Sex-specific analyses revealed different patterns. In male athletes, sprint times were largely similar, yet karate CoD performances varied substantially, suggesting that linear sprint speed alone does not explain variability in pre-planned CoD performance, consistent with prior evidence that technical and motor control factors are important in combat sports [26,27]. In female athletes, moderate correlations between sprint and karate CoD performances were observed; however, these findings should be interpreted cautiously, as they do not provide mechanistic evidence that linear speed contributes more to CoD ability in women. Associations with lower-limb power were low in both sexes, indicating that maximal horizontal strength is less influential on karate-specific pre-planned CoD performance than technical control, rapid repositioning, and coordination of movement sequences [1].

The karate CoD test demonstrated high test–retest reliability, indicating consistent measurement across trials. Discriminant validity analyses showed that higher-ranked athletes outperformed lower-ranked athletes, supporting the test’s utility for differentiating performance levels among elite practitioners. These results suggest that coaches can use the karate CoD test to monitor progression, identify potential movement asymmetries, and guide sport-specific training focused on multidirectional control and technical execution.

The pooled regression analysis revealed stronger associations between the karate CoD test and other performance measures compared with sex-specific analyses, likely reflecting increased variance when combining males and females [28]. However, pooled analyses may obscure sex-specific performance determinants, emphasizing the importance of interpreting results within sex-specific contexts for training and assessment purposes.

In summary, the present findings indicate that the karate-specific CoD test is a reliable and valid measure of pre-planned, sport-specific directional changes. The test demonstrated stronger associations with the Y CoD test than with the T-half test, and it was able to differentiate between higher- and lower-ranked athletes, supporting its potential utility for monitoring karate-specific CoD performance. However, correlations with linear sprinting and standing long jump were weak or inconsistent, suggesting that pre-planned CoD ability is not strongly predicted by general speed or lower-limb power, although this finding should be interpreted cautiously given the sample size, particularly for female athletes (n = 16).

Several limitations of the study should be acknowledged. First, the sample size was relatively small and exclusively composed of elite national team athletes, resulting in a highly homogeneous population. This limits the generalizability of the findings to lower-level, youth, or recreational karate practitioners. Second, the karate CoD test evaluated pre-planned change-of-direction ability only, without incorporating perceptual, decision-making, or reactive components, which are critical for true agility in combat sports. Third, the study relied on the best trial from three attempts for analysis, which may not fully represent consistent performance. Fourth, although technical correctness was monitored by certified evaluators, only two raters were involved, and formal inter-rater reliability was not assessed, introducing potential subjectivity. Fifth, longitudinal responsiveness to training was not examined, so it remains unknown whether improvements in test performance correspond to meaningful gains in competitive performance. Six, any interpretations of sex-specific patterns, such as the potential influence of sprint speed, should be made cautiously, as mechanistic evidence was not collected to support these observations. Taken together, these factors constrain the extent to which the karate CoD test can be used as a stand-alone profiling tool and highlight the need for complementary assessments and further validation in more diverse populations. Finally, while the newly developed karate CoD test was designed to simulate the multidirectional and technique-integrated nature of competitive karate, the present study provides only preliminary evidence regarding its ecological and tactical fidelity. The test’s movement structure was developed based on expert input and detailed examination of competition footage, which supports its face and content validity as a karate-specific assessment. However, in the absence of empirical data directly comparing test movements with those executed during actual bouts (e.g., through motion-capture or video-based biomechanical analyses), claims of full ecological validity remain tentative. Establishing such validity would require demonstrating that the test replicates not only the visual and tactical structure of in-match performance but also its kinematic and temporal characteristics. Future investigations should therefore aim to quantify these aspects to confirm the test’s representativeness of authentic karate performance.

From a practical perspective, the karate CoD test offers a structured and sport-specific means to assess pre-planned change-of-direction performance in youth or sub-elite karate athletes. However, its use should currently be considered exploratory rather than diagnostic or monitoring at the elite level. Given the study’s small and homogeneous sample, the absence of responsiveness data, and the lack of perceptual–decision-making components, the test should be interpreted as a preliminary tool for evaluating movement efficiency within controlled conditions. To obtain a more comprehensive understanding of an athlete’s physical and perceptual–motor capacities, it should be complemented with additional assessments—such as reactive agility drills, sprinting, jumping, and strength tests. Future research should examine the test’s sensitivity to performance changes, incorporate reactive and decision-making elements, and validate its application across different competitive levels before it can be used confidently for athlete profiling or longitudinal monitoring.

## 5. Conclusions

The present study indicates that the karate-specific change-of-direction (CoD) test demonstrates high test–retest reliability, supporting its consistency as a measurement tool. The test also showed a meaningful relationship with the Y-CoD test, which assesses structured multidirectional movements. However, it is important to emphasize that the karate CoD test primarily evaluates planned, pre-determined change-of-direction ability and does not incorporate reactive or decision-making components required for true agility. Associations with other motor abilities, including linear sprinting and lower-limb power, were weak or inconsistent, particularly in sex-specific analyses, suggesting that the test captures performance elements distinct from general physical qualities.

Overall, the karate CoD test appears to be a promising tool for monitoring planned, sport-specific CoD performance in elite karate athletes. Nevertheless, further validation in larger and more diverse populations, as well as assessment of its responsiveness to training interventions, is necessary. Practically, coaches may use the test to track karate-specific CoD performance, but it should be complemented with additional assessments, such as reactive agility drills, sprinting, jumping, or strength tests, to obtain a more comprehensive profile of athletes’ physical and perceptual-motor capabilities.

## Figures and Tables

**Table 1:** Consistency results of the karate change-of-direction test.

Parameter	Test (s)	Retest (s)	ICC [95% CI]	TEM (s)	TEM (%)	MDC_95_	MDC_95_ (%)
Karate CoD	7.65 ± 0.48	7.64 ± 47	0.97 [0.94–0.98]	0.16	2.13	0.44	5.89

## Data Availability

Data is contained within the article or Supplementary Material. The original contributions presented in this study are included in the article/Supplementary Material.

## Supplementary Materials

The following supporting information can be downloaded at https://www.mdpi.com/article/10.3390/jfmk10040417/s1.
